# Endoglin aggravates peritoneal fibrosis by regulating the activation of TGF-β/ALK/Smads signaling

**DOI:** 10.3389/fphar.2022.973182

**Published:** 2022-09-23

**Authors:** Qian Huang, Rui Xiao, Jing Lu, Yao Zhang, Liang Xu, Jie Gao, Jing Sun, Haiping Wang

**Affiliations:** ^1^ Department of Nephrology, Shandong Provincial Hospital, Shandong University, Jinan, China; ^2^ Department of Nephrology, Shandong Provincial Hospital Affiliated to Shandong First Medical University, Jinan, China

**Keywords:** endoglin, peritoneal fibrosis, angiogenesis, EMT, TGF-β/ALK/Smads

## Abstract

**Background:** Peritoneal fibrosis (PF) is an intractable complication in patients on long-term peritoneal dialysis (PD). Transforming growth factor-β (TGF-β) is a key pro-fibrogenic factor involved in PD-associated PF, and endoglin, as a coreceptor for TGF-β, plays a role in balancing the TGF-β signaling pathway. Here, we investigated whether endoglin could be a potential therapeutic target for PF.

**Methods:** *In vivo*, we established PF model in SD rats by daily intraperitoneal injection of peritoneal dialysis fluids (PDF) containing 4.25% glucose for 6 weeks and downregulated endoglin expression by tail vein injection of AAV9-ENG on day 14 to assess the effect of endoglin on peritoneal morphology and markers related to fibrosis, angiogenesis, and epithelial-mesenchymal transition (EMT). *In vitro*, we treated human peritoneal mesothelial cells (HPMCs) transfected with ENG siRNA in high glucose medium to explore the potential mechanism of endoglin in PF.

**Results:** Compared to control group, continuous exposure to biologically incompatible PDF induced exacerbated PF, accompanied by a significant increase in endoglin expression. Conversely, knockdown of endoglin ameliorated peritoneal injury characterized by increased peritoneal thickening and collagen deposition, angiogenesis, as well as EMT. Consistently, HPMCs cultured in high glucose medium underwent the EMT process and exhibited over-expression of fibronectin, collagen type I, vascular endothelial growth factor (VEGF), whereas these aforementioned alterations were alleviated after ENG siRNA transfection. In addition, we also found that ENG siRNA inhibited TGF-β-induced phosphorylation of Smad2/3 and Smad1/5/9 in HPMCs treated with high glucose (HG).

**Conclusion:** Our findings confirmed for the first time that endoglin exacerbated PF by regulating the activation of TGF-β/ALK/Smads signaling, which will provide a novel potential therapeutic target in PF.

## Introduction

Chronic kidney disease (CKD) has become a major public health problem in recent years due to the concomitant increase in the prevalence of its main associated risk factors, such as hypertension, diabetes and obesity (Lv and Zhang, 2019; Yang et al., 2020). As the disease continues to progress, the majority of CKD patients develop into end-stage renal disease (ESRD), a terminal stage requiring renal replacement treatment such as hemodialysis, peritoneal dialysis (PD), and kidney transplants. According to a recent report, clinical outcomes of PD are comparable to or better than hemodialysis, and PD patients can obtain a better quality of life (Li et al., 2017; Wong et al., 2018; Chuasuwan et al., 2020). However, PF limits the long-term application of PD in ESRD patients. Continuous exposure to bioincompatible, hypertonic PDF induces structural (fibrosis, angiogenesis, EMT) and functional (ultrafiltration failure) alterations in peritoneum, which eventually forces patients to withdraw from PD (de Lima et al., 2013; Zhou et al., 2016; Zhang et al., 2017; Balzer, 2020).

Mounting evidence indicates that PF pathophysiology involves fibrotic process itself, angiogenesis, and EMT (Balzer, 2020). The EMT process is a key mechanism contributing to PF. Mesothelial cells (MCs) that have undergone EMT can secrete pro-fibrogenic and angiogenic cytokines, inducing extracellular matrix (ECM) accumulation and vascularization (Selgas et al., 2006). The TGF-β signaling pathway is reported to play a predominant role in mediating PF, angiogenesis, and EMT. In HPMCs, prolonged exposure to PDF stimulates the production of TGF-β, which activates TGF-βRII and RI in turn. Subsequently, the TGF-β signaling pathway is transduced via phosphorylation of Smad2/3 and Smad1/5/8 and consecutive translocation of Smad4-Smad2/3 complex and Smad4-Smad1/5/8 into the nucleus. Various therapy strategies to attenuate PF by blocking the TGF-β/ALK/Smads signaling pathway have been reported. TGF-β1 receptor inhibitor GW788388 effectively inhibited TGF-β1-induced EMT in HPMCs, as well as improved PF and peritoneal membrane function in a PF mouse model (Lho et al., 2021). Similarly, blockade of thrombospondin-1 (TSP-1), a natural activator for TGF-β1, or siRNA-mediated knockdown of TGF-β both protected the peritoneal membrane from PDF-Induced damage (Yoshizawa et al., 2015; Jiang et al., 2019). Moreover, Smad3 knockout mice prevented PF and peritoneal dysfunction by attenuating EMT and reducing collagen formation (Duan et al., 2014).

Endoglin is a 180 kDa, homodimeric transmembrane glycoprotein that acts as an auxiliary receptor for ligands of the TGF-β superfamily. The TGF-β/endoglin/ALK/Smads signaling pathway has been reported to play a central role in several key cellular processes, including cell proliferation/migration, EMT, ECM synthesis and angiogenesis, making endoglin a star molecule for targeting tumorigenesis (Kwon et al., 2017; Kasprzak and Adamek, 2018; Paauwe et al., 2018; Jeng et al., 2021). There are a growing number of clinical trials testing the combination of TRC105, a chimeric immunoglobulin G1 monoclonal antibody that binds endoglin, with standard therapies in different tumor types, showing that TRC105 is a promising strategy against solid tumors (Choueiri et al., 2019; Liu et al., 2020; Choueiri et al., 2021). Since fibrosis, angiogenesis and EMT are also involved in the initiation and progression of PF, we speculated that targeting endoglin might also have a therapeutic effect in PF.

Hence, in this study, we investigated the role and mechanism of endoglin in PF, which will provide the evidence that supports the therapeutic targeting of endoglin as a novel clinical strategy for intervention of PF.

## Materials and methods

### Construction of AAV9-ENG

Recombinant adeno-associated virus (AAV9-ENG) and adeno-associated virus negative control (AAV9-NC) were constructed by Genomeditech (Shanghai, China). The single-stranded DNA oligo containing the interfering sequence was first synthesised, then annealed and paired to produce a double-stranded DNA oligo, which was then directly ligated into the enzymatically cleaved RNA-interfering adeno-associated viral vector through the enzymatic cleavage sites at both ends. The constructed adeno-associated virus vector and its helper packaging plasmid were co-transfected into AAV-293 cells. After 6–8 h of transfection, the medium was replaced with fresh medium and the Enhancing buffer was added. The cells were continued to be cultured for 72 h. The virus-rich cells and supernatant were collected after cell detachment, concentrated and purified to obtain a high concentration of virus concentrate. The viral titer was 1.10 × 10^13 V g/ml determined using quantitative PCR. The rat peritoneum was taken 4 weeks after AAV injection to evaluate the transfection effect by Western blot and immunohistochemical staining. In addition, since our adeno-associated viral vector carries the EGFP gene, its expression product fluoresces green under fluorescent microscopy. Therefore, fluorescence analyses of frozen sections can also be used to verify the ability of AAV9 vector to transduce peritoneum.

### Animal and experiments

The study protocol was approved by the principles of the Animal Care Ethics Committee of Shandong Provincial Hospital Affiliated with Shandong University. 32 male SD rats (6–8 weeks old), provided by Jinan Pengyue Experimental Animal Breeding Co., (Jinan, Shandong), were randomly divided into four groups (*n* = 8/group): Control group, PDF group, AAV9-NC+PDF group, AAV9-ENG+PDF group. To successfully establish PF animal model, we treated rats in PDF group with daily intraperitoneal injection of 4.25% glucose PDF (Baxter Healthcare Ltd., Deerfield, IL, United States) for 6 weeks. Control rats received an equal volume of normal saline (NS). Two weeks after the start of the experiment, AAV9-ENG (The viral titer was 1.10 × 10^13 V g/ml, 100 μl per rat), constructed by Genomeditech (Shanghai, China), was given to rats in the AAV9-ENG+PDF group by tail vein injection, while AAV9-NC was applied as a control to rats in the AAV9-NC group. Four weeks after AAV injection, we harvested anterior abdominal wall and fixed in 4% paraformaldehyde for pathological analysis, as well as stored omentum tissue under −80°C for protein expression analysis.

### Histopathologic evaluation

The peritoneum samples fixed in 4% paraformaldehyde underwent a series of standard procedures including successive dehydration in a graded alcohol series (75%, 85%, 95%, and 100%, v/v), transparency in dimethylbenzene and paraffin embedding, and then were sectioned into 5 µm thick slices. For histological examination, hematoxylin-eosin (HE) and Masson’s trichrome staining were conducted to show the pathological changes and peritoneum thickness according to standard protocols provided by the manufacturer (Sigma-Aldrich). We utilized ImageJ (National Institute of Health, Bethesda, MD), a computerized image analysis software, to evaluate PF by measuring the percentage of peritoneal membrane occupied by collagen fibers. In addition, peritoneum thickness was assessed by measuring the distance from the surface mesothelium to the upper limit of the muscular tissue with the aid of ImageJ software.

### Immunohistochemical investigation

The immunohistochemistry staining was performed to assess protein expression using the streptavidin–peroxidase immunohistochemical method. Paraffin-embedded tissue sections were dewaxed, hydrated and heat-treated with antigen recovery buffer (PH = 6). Subsequently, the sections were subjected to 3% hydrogen peroxide at room temperature for 10 min, then blocked with normal goat serum at room temperature for 15 min and incubated overnight at 4°C with primary antibodies as follows: Rabbit anti-endoglin (Proteintech, 10862-1-AP, 1:1000), rabbit anti-fibronectin (Proteintech, 15613-1-AP, 1:500), rabbit anti-collagen type I (Proteintech, 14695-1-AP, 1:500) and rabbit anti-CD31 (Abcam, ab182981, 1:1000). On the following day, the sections were washed with PBS and incubated with HRP-conjugated goat anti-rabbit IgG secondary antibodies for 15 min at room temperature, followed by incubation for 15 min with horseradish peroxidase–labeled streptavidin. After incubation for 5–10 min with DAB, the sections were counterstained with hematoxylin. Finally, the selected regions were captured under microscope (Leica, Germany).

### Cell culture and treatment

The human peritoneal mesothelial cell line HMrSV5 was purchased from Jennio Biotech Co., Ltd., (Guangzhou, China) and cultured in DMEM/F12 medium supplemented with 10% fetal bovine serum (FBS) and 1% penicillin/streptomycin at 37°C with a humid atmosphere containing 5% CO_2_. When the cells reached confluence, they were cultured in serum-free DMEM/F12 medium for overnight starvation prior to each experiment to render quiescent. Then, we stimulated HPMCs with DMEM/F12 containing various D-glucose concentrations [17 mM (0.31%), 83 mM (1.5%), 139 mM (2.5%), and 236 mM (4.25%)].

### siRNA transfection

We used siRNA targeting ENG (Genomeditech, Shanghai, China) to further identify the role of endoglin in PF. The target sequences were listed as follows: ENG siRNA: 5′-CAA​UGA​GGC​GGU​GGC​AAU-3′; NC siRNA: 5′-UUC​UCC​GAA​CGU​GUC​ACG​UTT-3′. According to the manufacturer’s standard protocol, ENG siRNA and NC siRNA were transiently transfected into cultured HPMCs with 60%–80% of confluence using Lipofectamine 2000 (Invitrogen, CA, United States). Then transfected cells were treated with high glucose for subsequent experiments.

### Cell counting kit-8 assay

The Cell Counting Kit-8 (CCK-8) (Elabscience, Wuhan, China) was used to detect the effect of different concentrations of HG on the proliferation of HPMCs. We seeded HPMCs in 96-well culture plates at a density of 3,000 cells per well. When the cells reached confluence, they were cultured in serum-free medium overnight under starvation conditions. We then treated them with different concentrations of HG (control, 1.5%, 2.5%, or 4.25%). After 48 h, 90 µl of fresh serum-free medium and 10 µl of CCK-8 were added to each well, followed by incubation for 2 h at 37°C. Finally, we measured the absorbance of each well at 450 nm with a microplate reader (EL340 BioTek Instruments, Hopkinton, MA, United States).

### Western blot analysis

We extracted total protein from the rat omentum tissue stored in −80°C and the cells treated with different experimental conditions by using RIPA lysis buffer containing protease inhibitors and phosphatase inhibitors. A BCA protein assay kit (Beyotime Biotechnology) was used to detect the total protein concentration so as to determine the volume of each sample for western blot analysis. We then mixed cell lysate with 4*SDS loading buffer and boiled for 10 min. Equal amounts of protein were separated by 10% sodium dodecyl sulfate-polyacrylamide gel electrophoresis (SDS-PAGE) and transferred to polyvinylidene difluoride (PVDF) membrane (Millipore, Billerica, MA, United States). The membranes were blocked with 5% skim milk for 1 h and incubated overnight at 4°C with the following primary antibodies: endoglin (Proteintech, 10862-1-AP, 1:1000), fibronectin (Proteintech, 15613-1-AP, 1:2000), collagen type I (Proteintech, 14695-1-AP, 1:1000), E-cadherin (Cell signaling technology, 3195T, 1:1000), N-cadherin (Proteintech, 22018-1-AP, 1:2000), vimentin (Abcam, ab92547, 1:1000), α-smooth muscle actin (α-SMA) (Proteintech, 14395-1-AP, 1:1000), VEGF (Proteintech, 19003-1-AP, 1:1000), TGF-β1 (Proteintech, 21898-1-AP, 1:1000), phospho-Smad2 (Cell signaling technology, 3108T, 1:1000), phospho-Smad3 (Abcam, ab52903, 1:2000), phospho-Smad1/5/9 (Cell signaling technology, 13820T, 1:1000), GAPDH (Abclonal, AC002, 1:5000). After washing with TBST buffer, the membranes were incubated with the relevant HRP-conjugated secondary antibodies at room temperature for 1 h. The bands obtained were visualized by using ECL reagent (Millipore, United States) and Amersham Imager 600 (GE, United States), and analyzed by means of ImageJ software.

### Immunofluorescence

HPMCs were seeded in 24-well dishes and treated with different interventions including HG and siRNA. Then cells were fixed with 4% Paraformaldehyde for 20 min, permeabilized in 0.2% Triton X-100 for 15 min, blocked with 5% BSA for 1 h at 37°C and incubated overnight at 4°C with primary antibodies, E-cadherin (Cell signaling technology, 3195T, 1:200) and α-SMA (Servicebio, GB111364, 1:200). Finally, the cells were incubated with the Alexa Fluor 488-conjugated secondary antibody (Invitrogen, CA, United States, 1:200) at 37°C for 1 h. After staining nuclei with DAPI (Beyotime Biotechnology), images were captured by fluorescence microscope (Leica, Germany).

### Statistical analysis

All experiments in this research were independently repeated at least three times. The data processed by GraphPad Prism 8.0.2 were presented as the mean ± SD. We evaluated statistical significance between two groups using Student’s *t*-test. To compare the difference among multiple groups, one-way ANOVA was used. *p* < 0.05 was considered as statistically significant.

## Results

### Endoglin was upregulated in peritoneal fibrosis rats

**FIGURE 1:**
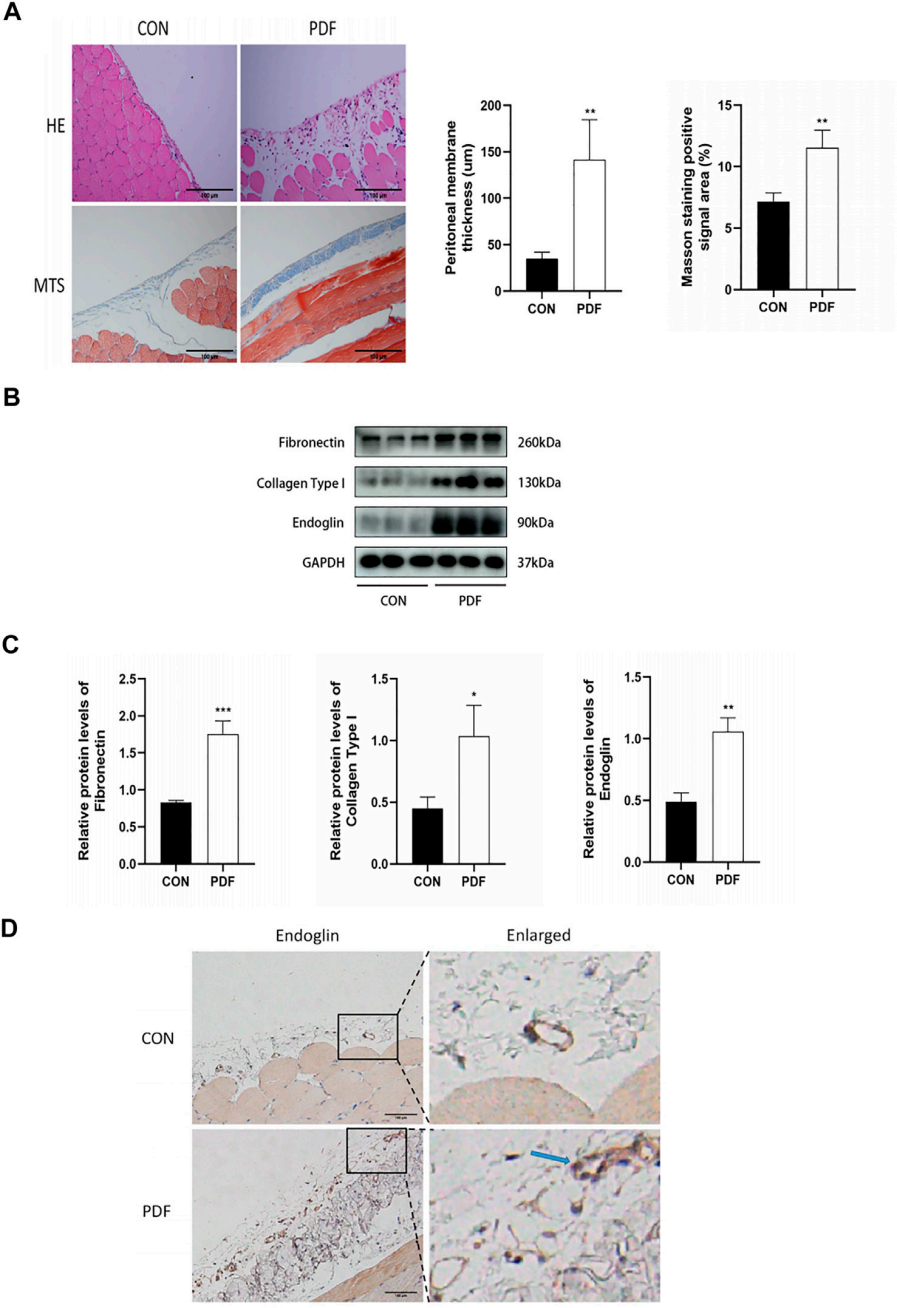
Endoglin was upregulated in PF rats. **(A)** Representative images of HE and Masson’s trichrome staining in peritoneum in control and PDF rats. Scale bar = 100 μm. Mean peritoneal membrane thickness and peritoneal fibrosis score in control and PDF rats. **(B)** Fibronectin, Collagen Type I and endoglin protein levels in control and PDF rats. **(C)** The graphs showed the quantitative analysis of Fibronectin, Collagen Type I and endoglin protein levels. **(D)** Representative immunohistochemical staining images of endoglin in control and PDF rats. The blue arrows indicate vascular endothelial cells. Scale bar = 100 μm. *n* = 6. All quantitative data are expressed as mean ± SD, *p < 0.05, **p < 0.01.

To determine the relationship between endoglin and PF, we firstly constructed PD-related PF model by daily intraperitoneal injection with 4.25% dextrose PDF. H&E staining and Masson trichrome staining revealed that, compared with control group, there was a significant increase in peritoneal membrane thickness and collagen fibrils accumulation in the PDF group (Figure 1A). The western blot results also showed increased expression of fibronectin and collagen type I in the PDF group (Figures 1B,C). We then detected the protein expression level of endoglin in the peritoneal anterior abdominal wall and omentum from rats treated with PDF. The immunohistochemistry staining and western blot assays indicated that the endoglin expression in PDF rats is higher than that in control group (Figures 1B–D). In general, we confirmed that endoglin was increased in rats with PD-related PF.

### Knockdown of endoglin attenuated peritoneal dialysis-induced peritoneal fibrosis

**FIGURE 2:**
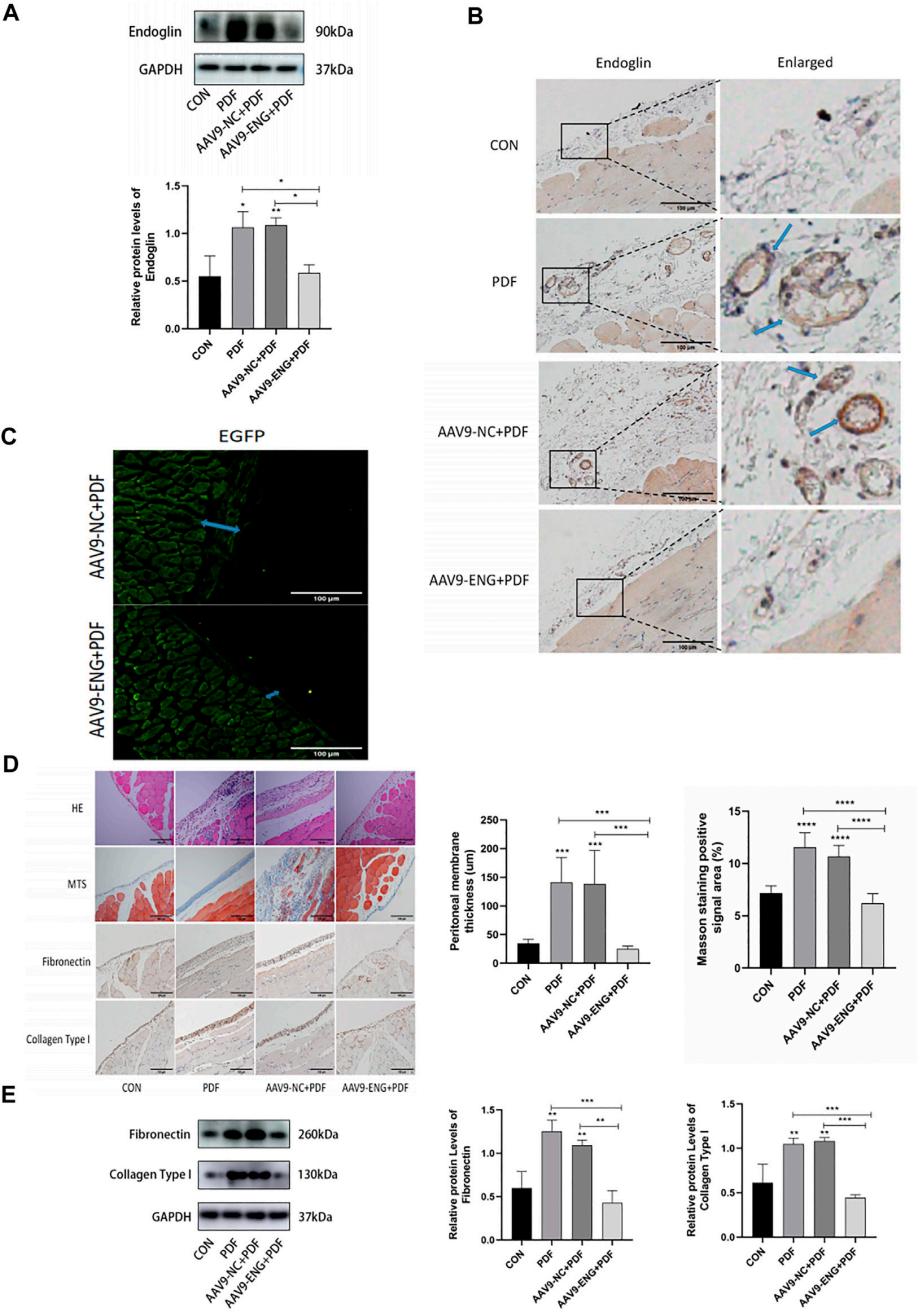
Knockdown of endoglin attenuated PD-induced PF. **(A)** Western blot analysis of endoglin protein levels in different groups of rats. **(B)** Representative immunohistochemical staining images of endoglin in different groups of rats. Scale bar = 100 μm. **(C)** Fluorescent images of frozen peritoneum sections. Scale bar = 100 μm. **(D)** HE, Masson’s trichrome staining and immunohistochemical staining images of Fibronectin and Collagen Type I protein in different groups. Scale bar = 100 μm. **(E)** Immunoblot analysis and corresponding statistical graph of Fibronectin and Collagen Type I. *n* = 6. All quantitative data are expressed as mean ± SD, *p < 0.05, **p < 0.01, ***p < 0.001, ****p < 0.0001.

To elucidate the role of endoglin in PF, we knocked down endoglin by injecting AAV9-ENG into tail vein of rats. The western blot results and immunohistochemical study showed that AAV9-ENG efficiently transduced into rat peritoneum and downregulated the increase of peritoneal endoglin expression induced by PDF (Figures 2A,B). We then evaluated whether endoglin deficiency would alleviate PD-induced PF. The results obtained from H&E staining, Masson trichrome staining and fluorescent images of frozen peritoneum sections exhibited improved peritoneal thickness and less collagen fiber deposition in AAV9-ENG+PDF group (Figures 2C,D). In addition, western blot results and immunohistochemical staining suggested that endoglin knockdown decreased the expression levels of fibrotic markers fibronectin, collagen type I (Figures 2D,E). Taken together, these results indicated that endoglin was involved in PF progression.

### Angiogenesis in endoglin knockdown rat

**FIGURE 3:**
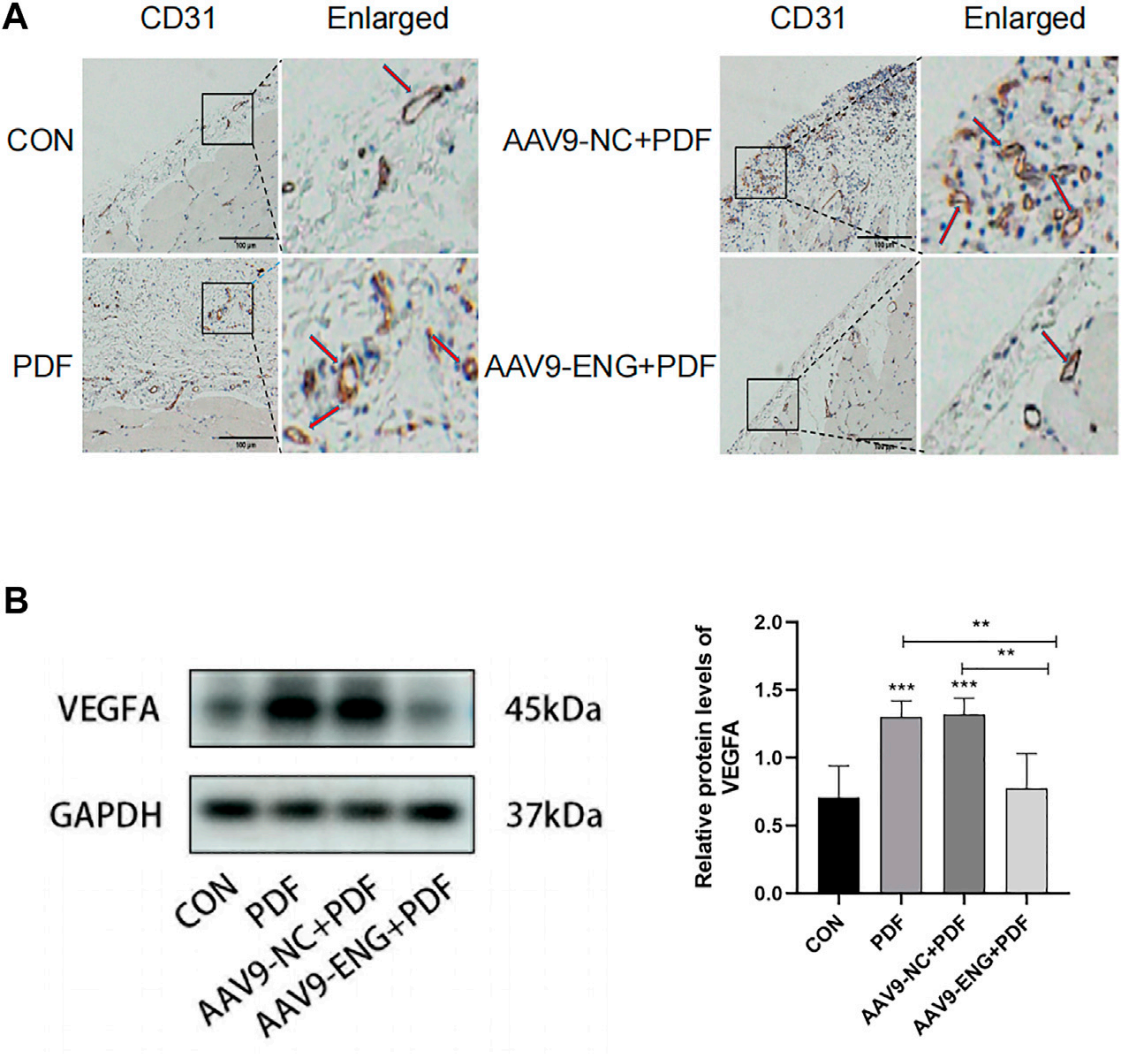
Angiogenesis in endoglin knockdown rat. **(A)** Representative immunohistochemical staining images of CD31 in different groups of rats. The red arrows indicate hyperplastic blood vessels. Scale bar = 100 μm. **(B)** VEGFA protein levels in different groups. *n* = 6. All quantitative data are expressed as mean ± SD, *p < 0.05, **p < 0.01, ***p < 0.001.

Since peritoneal angiogenesis is proven to play a key role in the development of PF, we explored whether endoglin deficiency could ameliorate angiogenesis by immunohistochemical staining for CD31, a marker of capillary sprouting. As shown in Figure 3A, exposure to PDF increased the number of CD31-positive vessels in the rat peritoneum, and the AAV9-ENG treatment inhibited this response. Moreover, considering the involvement of VEGF in peritoneal angiogenesis, we conducted western blot analysis for its expression. Compared with control, we observed a notably increased VEGF in PDF rats, which could be reversed by endoglin knockdown (Figure 3B). Together, these data demonstrated that endoglin deficiency could ameliorate peritoneal angiogenesis.

### Epithelial-mesenchymal transition in endoglin knockdown rat

**FIGURE 4:**
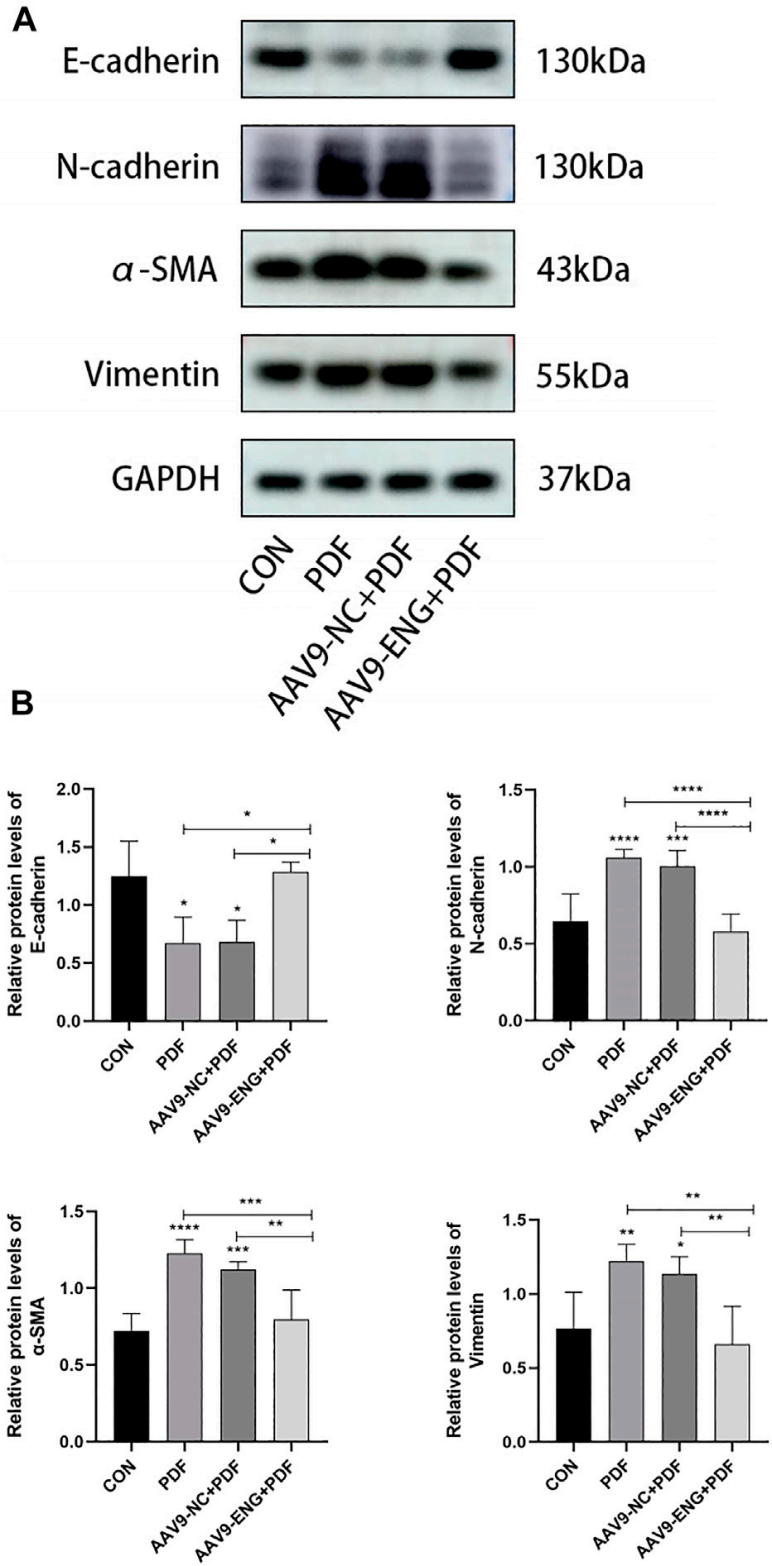
EMT in endoglin knockdown rat. **(A)** Western blot analysis of E-cadherin, N-cadherin, α-SMA and vimentin protein levels in different groups of rats. **(B)** The graphs showed the quantitative analysis of E-cadherin, N-cadherin, α-SMA and vimentin protein levels. *n* = 6. All quantitative data are expressed as mean ± SD, *p < 0.05, **p < 0.01, ***p < 0.001, ****p < 0.0001.

Given the critical role of EMT in PF, we evaluated the protein expression levels of EMT biomarkers E-cadherin, N-cadherin, α-SMA, and vimentin by western blot analysis to further investigate the impact of endoglin on EMT in PDF rats. We observed that PDF treatment induced downregulation of E-cadherin and upregulation of N-cadherin, α-SMA and vimentin compared with the control group, while endoglin deficiency reversed the above changes (Figures 4A,B). These results suggested that endoglin knockdown could reverse EMT process, which in turn acts to alleviate PF.

### Endoglin was upregulated in high glucose stimulated human peritoneal mesothelial cells

**FIGURE 5:**
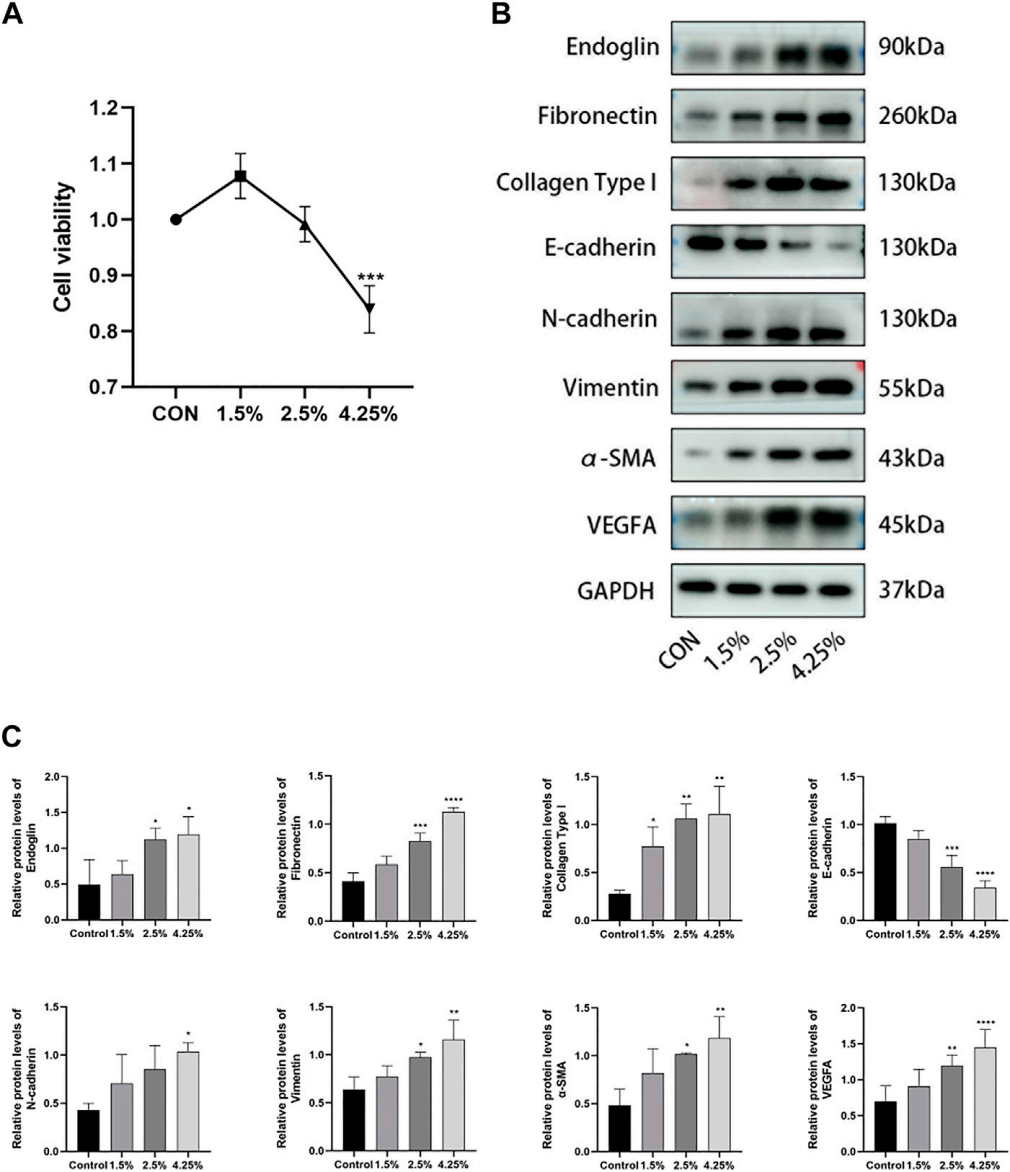
Endoglin was upregulated in HG stimulated HPMCs. **(A)** HPMCs were treated with different concentrations of HG (control, 1.5%, 2.5%, 4.25%) for 24 h, and CCK8 were performed to detect the viability of HPMCs. (Data are shown as mean ± SD, ***p < 0.001 compared with the control, *n* = 3). **(B)** Western blot detection of endoglin, Fibronectin, Collagen Type I, E-cadherin, N-cadherin, vimentin, α-SMA and VEGFA of peritoneum in different groups. GAPDH was used as a loading control. The results from the quantitative analysis are shown in **(C)**. (Data are shown as mean ± SD, *p < 0.05 compared with the control, **p < 0.01 compared with the control, ***p < 0.001 compared with the control, ****p < 0.0001 compared with the control).

To further identify the role of endoglin in PD-associated PF, we stimulated HPMCs with various concentrations of glucose (control, 1.5%, 2.5%, 4.25%) for 24 h. First, we assessed the impact of HG on cell proliferation by performing CCK-8 experiments and chose 2.5% glucose for subsequent experiments based on the results (Figure 5A). Then, we found that HG resulted in injury to HPMCs consistent with *in vivo* experiments, as evidenced by the downregulation of E-cadherin protein expression and the increased protein expression levels of N-cadherin, α-SMA, vimentin, fibronectin, collagen type I and VEGF (Figures 5B,C). By western blot analysis, we further detected endoglin expression and proved a significant increase of endoglin in HG-induced HPMCs (Figures 5B,C). Collectively, these results confirmed that increased endoglin protein level parallel changes in the expression levels of fibrosis, angiogenesis, and EMT-related proteins in HG-induced HPMCs.

### Downregulation of endoglin improved high glucose-induced injury in human peritoneal mesothelial cells

**FIGURE 6:**
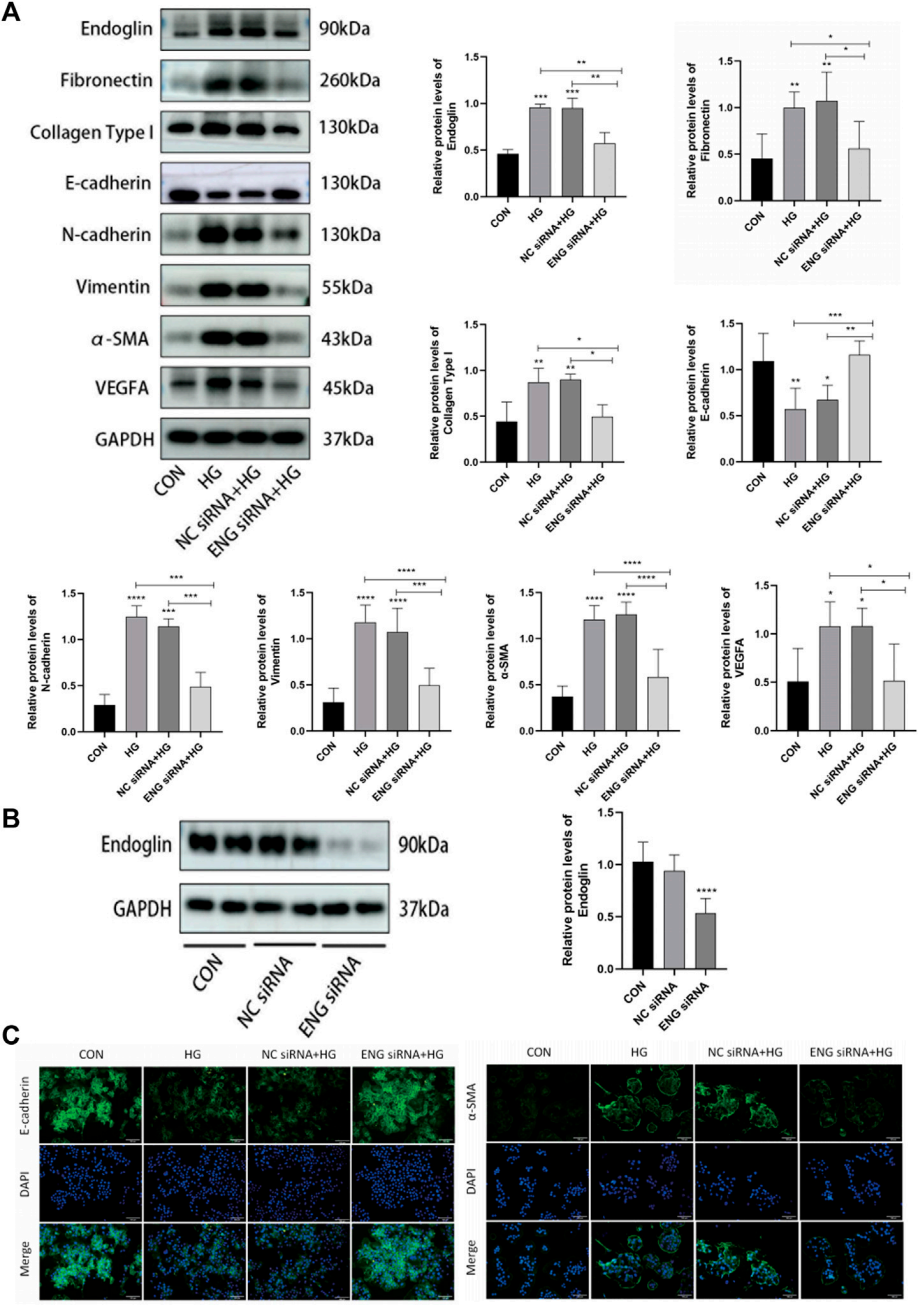
Downregulation of endoglin improved HG-induced injury in HPMCs. **(A)** Western blot detection of endoglin, Fibronectin, Collagen Type I, E-cadherin, N-cadherin, vimentin, α-SMA and VEGFA in HPMCs. GAPDH was used as a loading control. **(B)** HPMCs were treated with ENG siRNA or NC siRNA, the knockdown efficiency was examined by Western blot. (Data are shown as mean ± SD, ****p < 0.0001 compared with the control). **(C)** Representative immunofluorescence images of E-cadherin and α-SMA in HPMCs. Scale bar = 100 μm.

In order to further determine whether endoglin is involved in HG-induced HPMCs injury *in vitro*, we transfected ENG siRNA into HPMCs treated with HG to silence endoglin expression. Western blot results showed that endoglin deficiency resulted in a dramatic increase in E-cadherin protein levels and a significant decrease in N-cadherin, α-SMA, vimentin, fibronectin, collagen type I and VEGF protein expression levels in the HPMCs treated with 2.5% glucose alone (Figure 6A). The transfection efficiency of ENG siRNA was verified by western blot analysis (Figure 6B). Besides, Immunofluorescence staining also illustrated that ENG siRNA led to a notably increase of epithelial marker E-cadherin but decrease of mesenchymal marker α-SMA in the HPMCs after HG (2.5%) stimulation (Figure 6C).

### Endoglin deficiency inhibited the transforming growth factor-β/ALK/smads pathway involved in high glucose-induced human peritoneal mesothelial cells injury

**FIGURE 7:**
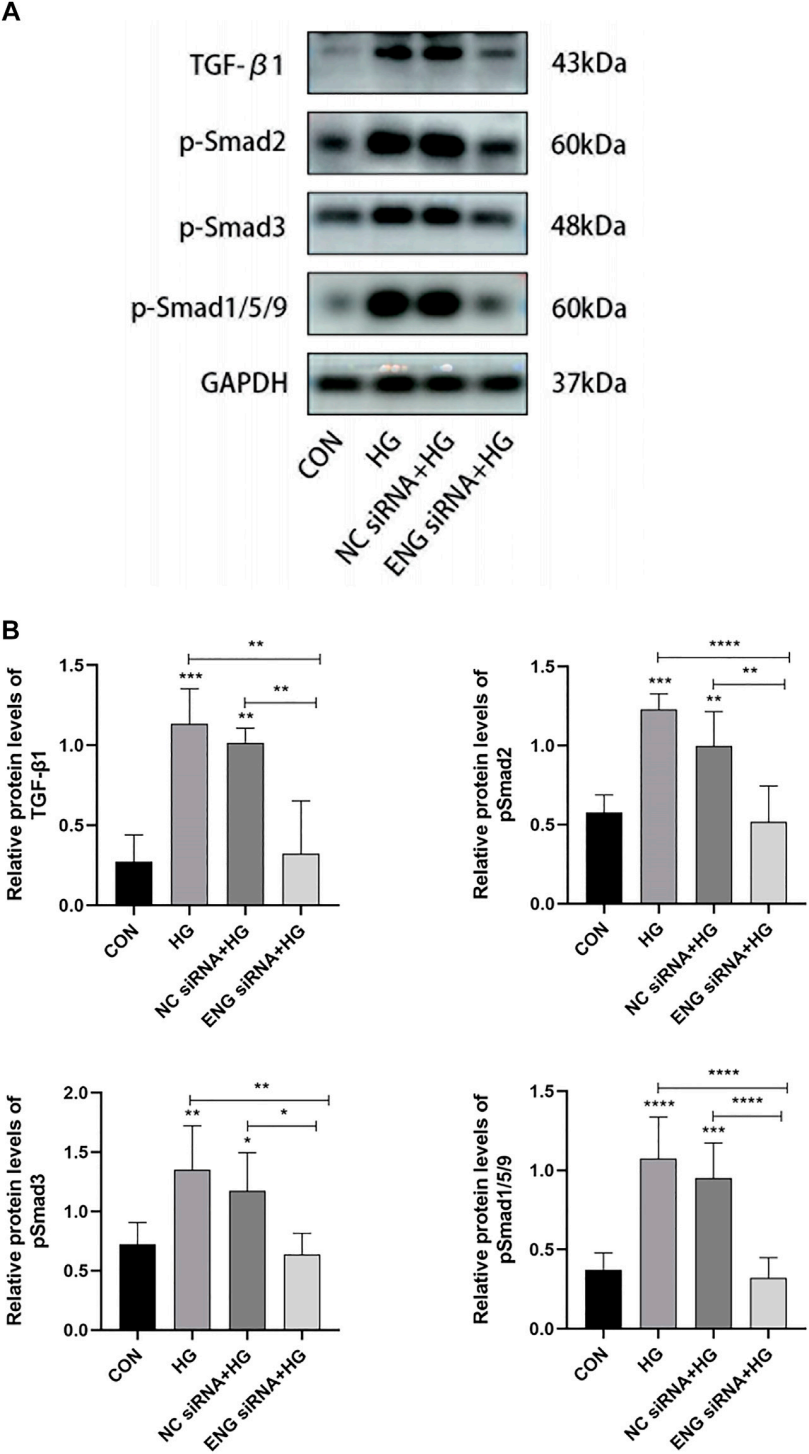
Endoglin deficiency inhibited the TGF-β/ALK/Smads pathway involved in HG-induced HPMCs injury. **(A)** Western blot analysis of TGF-β1, p-Smad2, p-Smad3 and p-Smad1/5/9 protein levels in different groups of HPMCs. The results from the quantitative analysis are shown in **(B)**. Data are shown as mean ± SD, *p < 0.05, **p < 0.01, ***p < 0.001, ****p < 0.0001.

Given the role of endoglin as TGF-β co-receptor, we performed western blot assay to elucidate whether endoglin mediated HG-induced HPMCs injury by the involvement of the TGF-β/ALK/Smads signaling pathway. Compared with control, HG (2.5%) treatment significantly elevated the protein expression level of TGF-β1 and increased the phosphorylation levels of Smad2, Smad3 and Smad1/5/9, indicating that the TGF-β/ALK/Smads pathway was activated in HG-induced HPMCs injury (Figures 7A,B). We then tested the protein levels of TGF-β1, pSmad2, pSmad3 and pSmad1/5/9 in endoglin knockdown HPMCs stimulated with HG (2.5%) and found that ENG siRNA inhibited the expression and phosphorylation levels of these factors in HG group (Figures 7A,B). Together, these data revealed that endoglin deficiency inhibited activation of the TGF-β/ALK/Smads pathway in HG-induced HPMCs injury.

## Discussion

PF is an unavoidable complication concomitant with long-term PD and is associated with poor prognosis in patients with ESRD. Based on the existing studies on the mechanism of PF, it can be summarized as a pathological process containing the crosstalk of fibrosis, angiogenesis and EMT (Balzer, 2020). TGF-β, a key fibrogenic factor, mediates fibrogenesis in PF. Endoglin acts as a co-receptor for TGF-β, exerting its effect by modulating TGF-β signal transduction. In this study, we evidenced that endoglin positively correlates with the development of PF by conducting *in vivo* and *in vitro* experiments. Moreover, our findings in the present study indicated that the HG/TGF-β/endoglin/ALK/Smads signaling pathway exerted an import influence on the pathogenesis of PF.

In recent years, mounting evidence indicates that endoglin is closely related to organ fibrosis. Several studies have identified that endoglin plays a pivotal role in cardiac fibrosis of different heart failure models and targeting endoglin attenuates cardiac fibrosis (Kapur et al., 2012; Lin et al., 2016; Shyu, 2017); Endoglin has also been found to be upregulated in renal biopsy samples from patients with diabetic nephropathy and in renal fibrosis models, including unilateral ureteral obstruction (UUO) and radiation-induced nephropathy (Rodriguez-Pena et al., 2002; Scharpfenecker et al., 2009; Munoz-Felix et al., 2014). However, to our knowledge, there are no studies on the role of endoglin in PF. In this study, we simulated PD process by daily intraperitoneal injection of PDF containing 4.25% HG, and found that endoglin was upregulated in the rat peritoneum treated with PDF. We also proved that HG injury induced fibrosis of HMPCs, accompanied by elevated expression of endoglin. To further validate our hypothesis, we downregulated endoglin expression both *in vivo* and *in vitro* and discovered that inhibition of endoglin partially reversed PF, suggesting that endoglin-targeting therapy may be effective in the treatment of PF.

It has been known that angiogenesis is a key event in the development of PF. Williams et al. (2002) performed the first comprehensive cross-sectional analysis of the morphological changes in the peritoneal membranes of patients undergoing PD and showed that the density of blood vessels per unit length of peritoneum was significantly higher for patients with peritoneal membrane function failure and was correlated with the degree of fibrosis. Furthermore, Zhu et al. (2021) proved that Tetramethylpyrazine (TMP) treatment inhibited peritoneal angiogenesis and also ameliorated peritoneal thickening and collagen deposition. These findings suggest that anti-angiogenic therapy may serve as a potential modality for preventing or reversing PF. Among all molecules that regulate peritoneal angiogenesis, VEGF as a strong pro-angiogenic factor binds to VEGFR expressed on the cell surface of vascular endothelial cells, stimulating the formation of new capillaries in the peritoneal membrane. Increased effective vascular surface area accelerates glucose uptake from the dialysate into the circulation, leading to a dissipation of glucose-driven osmotic pressure, which ultimately results in ultrafiltration failure (UF) (Aroeira et al., 2005; Saxena, 2008; de Lima et al., 2013; Shi et al., 2022). The selective high expression of endoglin on tumour vessels has led to widespread attention to it as a target for cancer therapy, reminding us whether anti-endoglin treatment could similarly improve peritoneal angiogenesis and thus reduce PF (Paauwe et al., 2016; Kasprzak and Adamek, 2018; Liu et al., 2020). In this study, we found that treatment with 4.25% dextrose PDF significantly triggers peritoneal angiogenesis as evidenced by the increased level of CD31, an endothelial cell marker, and VEGF. However, anti-endoglin therapy inhibited peritoneal angiogenesis. We also found that non-epithelioid mesothelial cells (MCs) secreted more VEGF than epithelioid MCs, which is consistent with previous reports that VEGF is expressed abundantly in PMCs, particularly in PMCs undergoing the EMT process (Selgas et al., 2006; Aroeira et al., 2007; Lopez-Cabrera, 2014). In contrast, endoglin deficiency decreased production of VEGF in HPMCs. Obviously, just as previous studies have shown an interconnection between endoglin and the VEGF signaling pathway, we speculate that the same is true for peritoneal angiogenesis during PD (Li et al., 2015; Jadhao et al., 2022). Although the relationship in detail needs to further elucidate, it is sure that endoglin may also be a therapeutic target for the treatment of peritoneal angiogenesis and UF.

EMT is a dynamic process during which epithelial cells acquire mesenchymal phenotypes and behavior following the downregulation of epithelial features (Lamouille et al., 2014; Singh et al., 2018; Dongre and Weinberg, 2019; Sha et al., 2019). As mentioned above, converging lines of evidence suggest that angiogenesis and fibrosis are closely interconnected through the EMT process, which makes it become a key mechanism to intervene the progression of PF. Endoglin has been proved to be involved in the development of cardiac valve and in the cell migration and invasion in endometriosis, ovarian cancer and clear cell renal carcinoma, all of which are achieved through regulation of the EMT process (Mercado-Pimentel et al., 2007; Hu et al., 2019; Chen et al., 2021; Zhang et al., 2021). In this study, we discovered that loss of endoglin expression mediated by either AAV9-ENG or ENG siRNA caused the reversal of EMT process and corresponding changes in the protein expression level of EMT markers including E-cadherin, N-cadherin, vimentin and α-SMA. Our findings suggest that endoglin may aggravates PF through modulation of EMT.

Among the molecular pathways involved in the PF, the TGF-β signaling pathway, comprising Smad-dependent and Smad-independent signaling, is one of the most hotly discussed (Balzer, 2020). In this study, we mainly focus on the canonical Smad signaling pathway that dominates in PF. This signaling pathway is initiated by binding of TGF-β or BMP ligands to TGF-β RII, which subsequently causes recruitment, transphosphorylation and activation of TGF-β RI (ALK5 or ALK1) and finally phosphorylates downstream Smad2/3 or Smad1/5/8, leading to signal transduction (Pomeraniec et al., 2015; Seystahl et al., 2015; Liu et al., 2020; Schoonderwoerd et al., 2020; Burghardt et al., 2021; Gonzalez Munoz et al., 2021). On the one hand, when the peritoneal membrane is continuously exposed to bioincompatible PDF, it is certain that the TGF-β1/ALK5/Smad2/3 signaling pathway is significantly activated in PMCs. In this study, we also demonstrated its activation in HG-stimulated HPMCs, as evidenced by the upregulation of TGF-β1 and phosphorylated Smad2 and Smad3 levels. At present, there exists controversy over whether the ALK1/Smad1/5/8 signaling pathway is activated. Loureiro et al. (2010) considered that MCs constitutively express BMP-7 and exhibit basal activation of BMP7-dependent Smad1/5/8 *in vitro*, and that these are downregulated in the presence of conventional PDF (Phillips and Fraser, 2010). In contrast, Zhang et al. (2020) demonstrated that TGF-β1 induced significant activation of the Smad1/5/9 pathway in HMrSV5 cells. In our study, the phosphorylation level of Smad1/5/9 was upregulated in HG-induced HPMCs. In conjunction with the account in studies related to organ fibrosis that the Smad1/5/9 signaling activity tends to be opposite to the pro-fibrotic, EMT-promoting activity possessed by the Smad2/3 signaling pathway, we suggest that activation of the Smad1/5/9 signaling pathway may be the result of antagonism of the Smad2/3 signaling pathway activation. Overall, the epithelial/mesenchymal status of PMCs is determined by the balance between Smad2/3 and Smad1/5/9 signaling and the Smad2/3 pathway favors the mesenchymal phenotype (Strippoli et al., 2016). We speculate that the level of phosphorylated smad1/5/9 measured by immunoblotting may be influenced by the time of stimulation, showing an increase, decrease or even no change, which requires further study. On the other hand, endothelial cells (ECs) undergo proliferation and migration to promote angiogenesis, which is seen as another essential step in the development of PF. Numerous studies have shown that TGF-β can activate both the ALK5 and ALK1 pathways in ECs. The activation of ALK1/Smad1/5/8 pathway promotes the proliferation and migration of ECs and thus exerts a pro-angiogenic effect, whereas the opposite is true for ALK5/Smad2/3 signaling pathway. Considering that TGF-β signaling pathway in ECs during angiogenesis has been reported in great detail, we did not further explore the ALK5 and ALK1 pathways in ECs exposed to conventional PDF in the present study.

Given the important role of balance between the Smad2/3 and Smad1/5/8 pathways mediated by TGF-β in the development of PF and the known role of endoglin in regulating TGF-β signaling, we explored how endoglin regulates TGF-β signaling in PMCs exposed to HG and thus exerts an inductive effect on PF in this study. Endoglin promotes TGF-β-induced Smad2/3 or Smad1/5/9 responses by interacting with ALK5 or ALK1 to form a TGF-β receptor signaling complex. Notably, the effect of endoglin on TGF-β signaling depends on the type of cell and tissue as well as the cellular context. In primary chondrocytes, endoglin enhances the ALK1/Smad1/5/8 pathway associated with catabolism and inhibits the ALK5/Smad2/3 pathway associated with anabolism, whereas endoglin may mediate cartilage formation via the ALK5/Smad2/3 pathway in mesenchymal stem cells (MSCs) expressing endoglin (Finnson et al., 2010; Bianchi et al., 2021). High endoglin expression stimulates the ALK1 pathway and indirectly inhibits ALK5 signaling, thus promoting the activation state of angiogenesis. In contrast, in the absence of endoglin, the TGF-β/ALK5 signaling pathway predominates and maintains quiescent the endothelium (Lebrin et al., 2004; Lebrin et al., 2005; Ollauri-Ibanez et al., 2017). In hepatocellular carcinogenesis (HCC), endoglin promotes ECs proliferation, migration and angiogenesis through activation of the ALK1/Smad1/5/8 pathway. Conversely, endoglin increases extracellular matrix (ECM) synthesis via the enhancement of ALK5/smad2/3 signal transduction (Jeng et al., 2021). In addition to the examples of differential regulation of ALK1/Smad1/5 and ALK5/Smad2/3 by endoglin already discussed above, O’Connor et al. (2007) reported that endoglin acts as a positive regulator of both ALK1-induced Smad1/5/8 activation and ALK5-induced Smad2/3 activation in bone marrow stromal cells. In the present study, we similarly found that siRNA-mediated endoglin knockdown inhibited HG-induced phosphorylation of smad2/3 and Smad1/5/9 in HPMCs, suggesting that endoglin is involved in HG-induced HPMCs injury through the canonical TGF-β pathway. The only plausible explanation for conflicting data is that there are two different alternatively spliced isoforms of endoglin, L-endoglin (L, long) and S-endoglin (S, short), which differentially modulate TGF-β signaling mediated by ALK1 and ALK5 (Perez-Gomez et al., 2005; Velasco et al., 2008; Blanco et al., 2015). L-endoglin enhanced the ALK1 pathway, while S-endoglin promoted the ALK5 pathway. Although some researchers have investigated the role of endoglin by generating stable transfectants of U937 cells or L6E9 myoblasts to overexpress the L- or S-endoglin isoforms, appropriate tools to clearly distinguish between them are still lacking as of today. Therefore, most studies published on endoglin barely have made little distinction between L- and S-endoglin. In the present study, we found that both the ALK1/Smad1/5/9 and ALK5/Smad2/3 signaling pathways were activated by endoglin, but did not further distinguish between L-endoglin and S-endoglin and investigate their respective effects on TGF-β signaling, which will be involved in our future studies.

In conclusion, we demonstrated that endoglin promotes PF, and downregulation of endoglin can relieve PF by suppressing EMT, fibrosis and angiogenesis. In addition, endoglin mediates peritoneal injury through canonical the TGF-β signaling pathway. Therefore, our findings suggest that targeting endoglin may be a therapeutic intervention to preserve the integrity of peritoneum for long-term PD patients.

## Data Availability

The original contributions presented in the study are included in the article/Supplementary Material, further inquiries can be directed to the corresponding authors.
