# Corrigendum: Endoglin aggravates peritoneal fibrosis by regulating the activation of TGF-β/ALK/Smads signaling

**DOI:** 10.3389/fphar.2024.1532797

**Published:** 2024-12-12

**Authors:** Qian Huang, Rui Xiao, Jing Lu, Yao Zhang, Liang Xu, Jie Gao, Jing Sun, Haiping Wang

**Affiliations:** ^1^ Department of Nephrology, Shandong Provincial Hospital, Shandong University, Jinan, China; ^2^ Department of Nephrology, Shandong Provincial Hospital Affiliated to Shandong First Medical University, Jinan, China

**Keywords:** endoglin, peritoneal fibrosis, angiogenesis, EMT, TGF-β/ALK/Smads

In the published article, there was an error in the **Funding** statement. Some information were erroneously omitted from the statement. The original statement stated: This research was funded by National Science Foundation for Young Scholars of China (Grant Nos. 81500584 and 81200530) and the Science and Technology Development Plan Program of Medicine and Health of Shandong Province (No. 202103050761). The correct Funding statement appears below.

